# Isotretinoin-associated exercise-induced anaphylaxis in a patient with birch pollinosis and soybean sensitization: case presentation and literature review

**DOI:** 10.1186/s13223-021-00604-8

**Published:** 2021-10-09

**Authors:** Anna Bamidis, Silke C. Hofmann

**Affiliations:** Department of Dermatology, Allergology and Dermatosurgery, Helios University Hospital Wuppertal, University Witten/Herdecke, Heusnerstr. 40, 42283 Wuppertal, Germany

**Keywords:** Soybean, Peanut, Exercise-induced anaphylaxis, Gly m 4, PR10-protein

## Abstract

**Background:**

Peanut and soybean allergies are listed as contraindication in the package leaflet of isotretinoin, a widely used treatment of acne vulgaris. Cross-reactivity between PR10-proteins in peanut, tree nuts, and soybean is particularly common in patients with birch pollinosis and may lead to anaphylactic reactions in sensitized patients after intake of soybean oil containing isotretinoin capsules.

**Case presentation:**

Here, we describe a young man with hazelnut and birch pollen allergy, who experienced exercise-induced anaphylaxis after isotretinoin intake on the third day of treatment. A complete allergy work-up was carried out, and sensitization to both peanut and soybean PR10-proteins was confirmed. However, oral provocation with isotretinoin remained negative in the absence of intense physical activity and longterm treatment was well tolerated.

**Conclusion:**

To our knowledge, this is the first report of an exercise-induced anaphylaxis due to isotretinoin therapy. Our literature review to assess tolerability of isotretinoin in patients allergic to peanut, tree nuts or soybean revealed only one other case of anaphylaxis in a cashew-nut allergic patient sensitized to soybean PR10-protein Gly m 4. While there are no reports on soybean allergic patients treated with isotretinoin, the vast majority of peanut or tree nut allergic patients tolerated isotretinoin. Therefore, we conclude that sensitization to soybean, peanut or tree nuts should not preclude isotretinoin therapy. Particular caution is however warranted in patients with soybean sensitization. Pre-treatment oral challenges with isotretinoin may be recommended and physicians should be aware of the potential role of cofactors.

## Introduction

Severe acne vulgaris greatly impairs quality of life of affected, mainly young patients, and is associated with anxiety, depression and discomfort [1]. Isotretinoin was approved in 1982 and has since proven to successfully induce prolonged remission or cure and to prevent scar formation.

Isotretinoin capsules contain a refined soybean oil with a low protein amount of < 10 µg/ml according to the European Medicines Agency [2] and may therefore trigger anaphylactic reactions in individuals with soybean or peanut allergy. Approximately 65% of patients with birch pollen allergy are sensitized to Bet v 1 cross-reactive PR-10 proteins found in pome and stone fruits, tree nuts (e.g. hazelnut), and legumes (e.g. soybean and peanut) [3, 4], although many of these patients are asymptomatic. Clinical manifestations including oropharyngeal symptoms (oral allergy syndrome), angioedema of the head region, or rarely anaphylaxis following consumption of Bet v 1- homologous allergens in e.g. soybean (Gly m 4), peanut (Ara h 8), or hazelnut (Cor a 1) represent the most frequent type of food allergy in Northern and Central Europe in adults [5].

A recent publication recommended to avoid isotretinoin therapy in all patients with detectable specific IgE-antibodies to soybean Bet v 1-homolog Gly m 4 or to soybean storage proteins Gly m 5 and Gly m 6 [6]. This recommendation was based on a case report that described facial and pharyngeal angioedema after isotretinoin intake in a cashew nut allergic patient with IgE antibodies specific to soybean Gly m 4 [7].

Here, we report a case that highlights the challenges involved in prescribing isotretinoin in patients with soybean sensitization, and present a literature review of all published cases of patients with relevant food allergies undergoing isotretinoin therapy.

## Case report

Therapy of acne conglobata with 20 mg isotretinoin / d was initiated in December 2019 in a 17-year-old male patient with a history of birch pollen allergy, an oral allergy syndrome to hazelnut and apples, and a history of angioedema and dyspnoea after intake of sesame-containing Halva. The patient had previously tolerated peanut, but was not aware of prior consumption of soybean containing foodstuffs. On the third day of treatment the patient went jogging without having eaten anything or taken any other medication in the past 4 h. He developed dyspnoea, angiooedema, urticaria, and abdominal cramps during exercise approximately 20 min after isotretinoin intake, and was treated with oral steroids and antihistamines. He had not eaten any products containing traces of soybean or peanut on that day.

Laboratory results demonstrated sensitization to the major birch pollen allergen Bet v 1 (> 100 kU/l, norm < 0.35), the homologous peanut allergen Ara h 8 (> 100 kU/l) and to the peanut lipid transfer protein Ara h 9 (10.8 kU/l). In addition, IgE to the Bet v 1-homologue in soy (Gly m 4, 95.0 kUA/l) was strongly elevated. Baseline tryptase level was normal (4.1 µg/l) (Table 1).

**Table 1:** Characteristics of our patient and of published cases with isotretinoin therapy and relevant food allergy

Patients	Relevant allergy history	Prick test isotre-tinoin	Oral provocation isotretinoin	Tolerance of isotretinoin therapy	Total serum IgE	IgE to soybean allergens	Prick soy-bean	IgE to peanut allergens	Prick peanut	References
n = 2	Anaphylaxis not specified (peanut)		No reaction	Tolerated						Walsh et al. [15]
n = 6	Severe anaphylaxis (peanut)	Neg	No reaction	Tolerated			+		+	Spierings et al. [21]
n = 1	Allergy to cashew nut			Facial angioedema after 1st dose, throat swelling on day 3		Gly m 4 1.38 kU/l				Alden et al. [7]
n = 4	Allergy to peanut			Tolerated		Gly m 4 neg Gly m 5 neg Gly m 6 neg	−			Alden et al. [7]
n = 1	Peanut elimination since childhood due to IgE detection	Neg	No reaction	Tolerated	4229 kU/l	Soybean extract 0.39 kU/l	+	Peanut extract 35.7 kU/l Ara h 2 7.97 kU/l Ara h 8 3.72 kU/l	+	Alvarez-Arango et al. [20]
n = 1	Urticaria, facial angioedema, dyspnoea (peanut)	Neg	no reaction	Tolerated	959 kU/l	Soybean extract neg		Peanut extract 3.93 kU/l Ara h 2 0.71 kU/l	+	Pierret et al. [16]
n = 1	Nausea, throat tightness (peanut)	Neg	No reaction	Tolerated	817 kU/l			Ara h 2 4.12 kU/l Ara h 8 9.74 kU/l Ara h 9 neg	+	Hulstaert et al. [17]
n = 1	Anaphylaxis to peanut	Neg	No reaction	Tolerated					+	Paugam et al. [18]
n = 1	Facial angio-edema, pruritus, dyspnoea (peanut)	Neg	No reaction	Tolerated	915 kU/l	Gly m 4 neg Gly m 5 6.82 kU/l Gly m 6 12.7 kU/l	+	Ara h 2 > 100 Ara h 8 neg Ara h 9 neg	+	Denorme et al. [13]
n = 11	Allergy to peanut (n = 10), to cashew (n = 1)			Tolerated		Gly m 4 neg Gly m 5 neg Gly m 6 neg	−			Alden et al. [6]
n = 7	Allergy to peanut or tree nut	Neg	No reaction	Tolerated						Ostlere et al. [2]
n = 1	Allergy to peanut			Tolerated		Soybean extract neg	−		+	Humphrey et al. [19]
n = 1	Oral allergy syndrome to hazelnut, dyspnoea after sesame	Neg	No reaction	Exercise-induced anaphylaxis (°2), however tolerance in absence of cofactors	2903 kU/l	Gly m 4 95.0 kU/l Gly m 5 neg Gly m 6 neg	+	Ara h 2 neg Ara h 8 > 100 Ara h 9 10.8	+	Present case

Prick tests were read after 20 min and classified as follows: wheal of 3 mm in diameter (+), 4 mm (++), 5 mm (+++), and ≥ 6 mm (++++). The patient showed positive results for commercial extracts (ALK-Abello, Hamburg, Germany) of birch pollen (++++), peanut (+++), and soybean (+++), but prick-to-prick-testing with isotretinoin 20 mg capsules remained negative. An oral provocation with an isotretinoin 20 mg capsule was performed at the hospital and well tolerated. The patient refused an exercise challenge for diagnostic confirmation due to risk of anaphylaxis.

The role of exercise and other potential cofactors in triggering food-dependent anaphylaxis was explained to the young man. He was advised to carry an epinephrine autoinjector and to avoid soybean-containing foodstuffs in the presence of potential cofactors. Since he had never reacted to peanuts, we did not recommend a peanut-free diet. Subsequently, the patient was able to continue isotretinoin therapy in the absence of physical exercise.

## Discussion

In Central Europe, birch pollen allergy has the highest prevalence of all types of pollinosis with 6–17% of the population being sensitized to birch [5]. IgE-crossreactions between birch pollen major allergen Bet v 1 and the structurally very similar soybean PR10-protein Gly m 4 leads to the most common type of soybean allergy in this region. Approximately 10% of birch pollen allergic individuals develop mostly mild clinical manifestations after consumption of tofu, soy milk, or other soy-containing foodstuffs [8]. However, Gly m 4-sensitization may rarely induce severe anaphylactic reactions [4, 9].

Related Bet v 1-homologs are also present in peanut and various tree nuts or drupes (e.g. hazelnut, walnut, cashew, and almond) [10] usually leading to oropharyngeal symptoms. Anaphylactic reactions to these plant-derived foodstuffs may occur after ingestion of very high amounts of causative foodstuffs or due to presence of cofactors, e.g. timely physical exercise, intake of non-steroidal anti-inflammatory drugs (NSAIDs), proton pump inhibitors or alcohol, acute infections or stress. Cases of food-dependent exercise-induced anaphylaxis (FDEIA) after consumption of almonds, hazelnut, or soybean have been reported [11, 12].

Since isotretinoin is solubilized in refined soybean oil, the manufacturers discourage prescription in patients with soybean or peanut allergy according to the package leaflet [13]. However, refined soybean oil contains only very low amounts of soy proteins and minimal IgE-binding is to be expected [14].

We performed a literature review to identify all published cases with isotretinoin therapy and allergy to soybean, peanut, tree nuts, or birch. In line with the manufacturers instructions, the literature review did not identify any published cases of soybean allergic patients treated with isotretinoin. However, various publications suggest that a vast majority of peanut and tree nut sensitized patients, even those with clinically manifest allergy, benefit from isotretinoin therapy without experiencing allergic reactions (Table 1) [2, 6, 7, 15–19]. There were no publications describing prescription of isotretinoin specifically in birch pollen allergic patients.

Including our patient, up to now only 2 cases of anaphylaxis after isotretinoin intake have been reported in patients with previously known allergy to tree nuts, either cashew nut or hazelnut [7]. Both patients were sensitized to the soybean PR10-allergen Gly m 4 but had no clinically relevant soybean or peanut allergy.

Alden et al. recently proposed to determine IgE against soybean allergens Gly m 4, Gly m 5, and Gly m 6 and to perform prick tests with soybean in all patients with a positive history of peanut or tree nut allergy before isotretinoin therapy induction [6]. They considered patients with detectable sensitization to soybean unsuitable for isotretinoin therapy.

In contrast to this prior publication, our literature review demonstrates that isotretinoin was tolerated in eight patients with sensitization to soybean and a clinically manifest peanut allergy [13, 20, 21]. Soybean sensitization was confirmed by skin prick tests in the majority of these cases. Molecular allergy diagnostics was only performed in one case of a peanut allergic patient with isolated sensitization to stabile soybean storage proteins Gly m 5 and Gly m 6 who tolerated isotretinoin therapy [13].

Our case indicates that despite initial tolerance, physical activity may enhance the risk of isotretinoin-induced anaphylaxis in patients with tree nut allergy and sensitization to soybean. In general, food-dependent exercise-induced anaphylaxis (FDEIA) is a potentially life-threatening event, most frequently caused by wheat, nuts, and shellfish [22]. Exercise, non-steroidal anti-inflammatory drugs (NSAIDs), alcohol, and other cofactors may elicit anaphylactic reactions in sensitized individuals despite consumption of sub-threshold amounts of allergen by modulating mucosal permeability [23]. In soybean allergic patients, FDEIA has only rarely been reported in the context of sensitization either to storage proteins or to Gly m 4 [24–26]. A recent retrospective multicentre study revealed that intake of soybean-based drinks (e.g. soy milk), fasting, and excess of allergen are additional cofactors associated with an increased risk of triggering anaphylaxis in patients strongly sensitized to PR10-proteins [27].

To our knowledge, our patient represents the first case of an exercise-induced anaphylaxis due to traces of soybean in isotretinoin capsules. Although a causal relationship could not be proven due to the patients refusal of an exercise challenge, the fact that the patient denied consumption of any food or having taken any other medication prior to jogging, the temporal relationship, and the strong sensitization to Gly m 4 support the diagnosis of an isotretinoin-associated exercise-induced anaphylaxis.

This case therefore illustrates that atopic patients with birch pollen allergy, pollen-food-syndrome to tree nuts or peanut, and sensitization to soybean may be at risk for isotretinoin-induced allergy triggered by cofactors. However, these patients should not be prevented from accessing isotretinoin therapy, as most will tolerate it without difficulty. Based on this case and available literature, we suggest screening at risk patients for sensitization to soybean (including determination of specific IgE to Gly m 4) and to peanut. Patients with positive test results can be offered an oral provocation with isotretinoin under medical supervision. If tolerated, they can continue therapy, but may be counselled regarding potential cofactors and be advised to avoid intensive physical exercise within 4 h of the dose.

## Conclusion

Dermatologists as well as allergologists should elicit a history regarding soybean, peanut or tree nut allergy before isotretinoin prescription, perform pre-treatment oral challenges with isotretinoin in soybean sensitized individuals, and should be aware of a putative role of cofactors in triggering anaphylaxis after isotretinoin intake.

## Data Availability

All data analysed are included in this published article.

## References

[1] Altunay IK, Özkur E, Dalgard F, Gieler U, Tomas-Aragones L, Lien L (2020). Psychosocial aspects of adult acne: data from 13 European countries. Acta Derm Venereol.

[2] Ostlere L, Bansal A (2020). Management of soya and peanut allergic patients taking oral isotretinoin. Comment on: four-year data from use of the nut and soya testing protocol before treatment with isotretinoin and alitretinoin. Clin Exp Dermatol.

[3] Vieths S, Scheurer S, Ballmer-Weber B (2020). Current understanding of cross-reactivity of food allergens and pollen. Ann NY Acad Sci.

[4] Mittag D, Vieths S, Vogel L, Becker WM, Rihs HP, Helbling A (2004). Soybean allergy in patients allergic to birch pollen: clinical investigation and molecular characterization of allergens. J Allergy Clin Immunol.

[5] Kleine-Tebbe J, Ballmer-Weber B, Breiteneder H, Vieths S, Kleine-Tebbe J, Jakob T (2017). Bet v 1 and its homologs. Molecular allergy diagnostics.

[6] Alden K, Chowdhury MM, Williams PE, Kalavala M (2020). Four-year data from use of the nut and soya testing protocol before treatment with isotretinoin and alitretinoin. Clin Exp Dermatol.

[7] Alden K, Chowdhury MM, Williams PE, Kalavala M (2016). Protocol for investigation of possible soy allergy in patients being considered for treatment with isotretinoin or alitretinoin. Clin Exp Dermatol.

[8] Ballmer-Weber K, Vieths S (2008). Soy allergy in perspective. Curr Opin Allergy Clin Immunol.

[9] Berneder M, Bublin M, Hoffmann-Sommergruber K, Hawranek T, Lang R (2013). Allergen chip diagnosis for soy-allergic patients: Gly m 4 as a marker for severe food-allergic reactions to soy. Int Arch Allergy Immunol.

[10] Bastiaan-Net S, Batstra MR, Aazamy N, Savelkoul HFJ, van der Valk JPM, van Wijk RG (2020). IgE cross-reactivity measurement of cashew nut, hazelnut and peanut using a novel IMMULITE inhibition method. Clin Chem Lab Med.

[11] Senders AS, Oropeza AR, Kristensen B, Eller E, Kjaer HF, Bindslev-Jensen C (2018). Food-dependent exercise-induced anaphylaxis due to almond in a PR-10-sensitized patient. J Allergy Clin Immunol Pract.

[12] Baek CH, Bae YJ, Cho YS, Moon HB, Kim TB (2010). Food-dependent exercise-induced anaphylaxis in the celery-mugwort-birch-spice syndrome. Allergy.

[13] Denorme P, Schrijvers R, Van Hoeyveld E, Verfaillie S, Bullens D, Morren MA (2019). Isotretinoin in severe peanut- and soy-allergic patients: is it safe or not?. J Investig Allergol Clin Immunol.

[14] Errahali Y, Morisset M, Moneret-Vautrin DA (2002). Allergen in soy oils. Allergy.

[15] Walsh SA, Holme SA (2008). Acne successfully treated with acitretin in a patient with peanut allergy. Reply Br J Dermatol.

[16] Pierret L, Grosber M, Gutermuth J (2016). Is isotretinoin treatment safe in patients with known peanut allergy?. J Eur Acad Dermatol Venereol.

[17] Hulstaert E, Van Autryve E, Temmerman L (2016). Is isotretinoin treatment safe in patients with known peanut allergy?. Reply J Eur Acad Dermatol Venereol.

[18] Paugam C, Saint-Jean M, Colas L, Bernier C, Dréno B (2018). Isotretinoin treatment and peanut allergy: a new case report and review of the literature. J Eur Acad Dermatol Venereol.

[19] Humphrey VS, Phillips SM, James WD (2020). Is oral isotretinoin treatment contraindicated for patients with known peanut allergies?. JAAD Case Rep.

[20] Alvarez-Arango S, Hou A, Lowes MA, Jerschow E (2016). Isotretinoin treatment in a patient with known peanut allergy and positive IgE test results for soybean. Ann Allergy Asthma Immunol.

[21] Spierings NM, Natkunarajah J, Bansal A, Ostlere L (2015). Should we be prescribing isotretinoin to patients with peanut allergies?. Clin Exp Dermatol.

[22] Hofmann SC, Fischer J, Eriksson C, Bengtsson Gref O, Biedermann T, Jakob T (2012). IgE detection to α/β/γ-gliadin and its clinical relevance in wheat-dependent exercise-induced anaphylaxis. Allergy.

[23] Wölbing F, Fischer J, Köberle M, Kaesler S, Biedermann T (2013). About the role and underlying mechanisms of cofactors in anaphylaxis. Allergy.

[24] Hayashi M, Pawankar R, Yamanishi S, Itoh Y (2020). Food-dependent exercise-induced anaphylaxis to soybean: Gly m 5 and Gly m 6 as causative allergen components. World Allergy Org J.

[25] Izadi N, Rabinovitch N (2019). Food-dependent exercise-induced anaphylaxis to soybean. J Allergy Clin Immunol Pract.

[26] Adachi A, Horikawa T, Shimizu H (2009). Soybean beta-conglycinin as the main allergen in a patient with food-dependent exercise-induced anaphylaxis by tofu: food processing alters pepsin resistance. Clin Exp Allergy.

[27] Asero R, Ariano R, Aruanno A (2021). Systemic allergic reactions induced by labile plant-food allergens: seeking potential cofactors. A multicenter study. Allergy.

